# A National Baseline Prevalence Survey of Schistosomiasis in the Philippines Using Stratified Two-Step Systematic Cluster Sampling Design

**DOI:** 10.1155/2012/936128

**Published:** 2012-02-15

**Authors:** Lydia Leonardo, Pilarita Rivera, Ofelia Saniel, Elena Villacorte, May Antonnette Lebanan, Bobby Crisostomo, Leda Hernandez, Mario Baquilod, Edgardo Erce, Ruth Martinez, Raman Velayudhan

**Affiliations:** ^1^College of Public Health, University of the Philippines Manila, 625 Pedro Gil Street, Manila 1000, Philippines; ^2^Philippine Health Insurance Incorporated, Quezon City, Philippines; ^3^National Center for Disease Prevention and Control, Philippine Department of Health, Santa Cruz, Manila, Philippines; ^4^World Health Organization, San Lazaro Compound, Santa Cruz, Manila, Philippines

## Abstract

For the first time in the country, a national baseline prevalence survey using a well-defined sampling design such as a stratified two-step systematic cluster sampling was conducted in 2005 to 2008. The purpose of the survey was to stratify the provinces according to prevalence of schistosomiasis such as high, moderate, and low prevalence which in turn would be used as basis for the intervention program to be implemented. The national survey was divided into four phases. Results of the first two phases conducted in Mindanao and the Visayas were published in 2008. Data from the last two phases showed three provinces with prevalence rates higher than endemic provinces surveyed in the first two phases thus changing the overall ranking of endemic provinces at the national level. Age and sex distribution of schistosomiasis remained the same in Luzon and Maguindanao. Soil-transmitted and food-borne helminthes were also recorded in these surveys. This paper deals with the results of the last 2 phases done in Luzon and Maguindanao and integrates all four phases in the discussion.

## 1. Introduction

Schistosomiasis is a water-borne trematode infection that is endemic in 76 countries 46 of which are in Africa. About 207 M people are infected with 120 M people showing symptoms and 20 M severely ill [1]. In the Philippines, the disease is endemic in 28 out of 81 provinces distributed in 12 regions with a total population of 12 M exposed to the disease [2]. The infection affects almost the whole of the Mindanao region, the eastern part of the Visayas, and a few provinces in Luzon [3].

Surveys on a national scale have been scarce in the past. Most of these surveys were conducted in endemic areas using active and passive case finding. The lack of a well-defined study design resulted in overrepresentation or underrepresentation in the outcome of these surveys (Philippine Department of Health, unpublished data).

Mass treatment using praziquantel has been a mainstay in the control program for schistosomiasis. The subsequent reduction in prevalence in many endemic areas resulting from mass treatment has encouraged the Philippine Department of Health to pursue further its goal in schistosomiasis control such as to press further down the prevalence in some areas and to even push for elimination in some areas.

Streamlining of the program would be based on stratification of provinces into high prevalence, moderate prevalence, and low prevalence status. Accurate stratification is dependent on a baseline prevalence survey using an appropriate sampling technique that would generate updated data on the situation of schistosomiasis in the country [4]. Intervention programs are contingent on the disease status such that high burden areas may have to employ mass drug administration to dramatically bring down the prevalence and intensity of infection and subsequently reduce morbidity. Areas that have reached elimination status would require closer monitoring and surveillance [5].

The necessity for a baseline prevalence survey brought together several stakeholders to discuss and reach a consensus on various crucial issues attendant in a schistosomiasis survey such as sample size, sampling design, number of Kato-Katz thick smear examinations, and quality assurance. These meetings concluded with the implementation of a national baseline prevalence survey for schistosomiasis that has for its goal the stratification of schistosomiasis endemic areas into high, moderate, and low prevalence areas [5]. The study was divided into several phases spread out in four years from 2005 to 2008. Results of the first two phases were published in a paper in 2008 [5].

This paper deals with the results from phases 3 and 4 conducted in Luzon and Maguindanao, respectively. Maguindanao is a province in the Autonomous Region of Muslim Mindanao (ARMM) which was missed during the Mindanao survey in 2005. The need to include this province in the survey could not be overemphasized given that the objective of the national survey is to stratify schistosomiasis endemic provinces into high prevalence, moderate prevalence, and low prevalence areas. Past data show that Maguindanao is included among the high prevalence areas, hence the separate phase to obtain the most recent information about the status of the disease.

The paper integrates results of all four phases in the discussion to present a complete account of the national baseline prevalence survey conducted in 2005–2008.

## 2. Materials and Methods

### 2.1. Sampling Design

As in the first two phases, the sampling design used in these last two phases was stratified two-stage systematic cluster sampling [6]. The Luzon survey took place in September 1, 2007 to November 30, 2007 while the Maguindanao survey started in April 2008 and was completed at the end of August 2008.

The survey covered all the eight regions in Luzon and made sure that the three endemic provinces are included, namely, Sorsogon (Region V), Mindoro Oriental (Region IV-B), and Cagayan Valley (Region II) which was established as an endemic province only in 2002.

The nonendemic provinces were randomly selected. The provinces were used as primary sampling units and the barangays as secondary sampling units. The households were systematically selected and the households represent the clusters. The provinces surveyed and the regions where they belong are as follows.

Cordillera Administrative Region: Apayao,
Region I: Ilocos Sur,
Region II: Cagayan Valley,
Region III: Aurora,
Region IV-A: Rizal,
Region IV-B: Mindoro Oriental,
Region V: Sorsogon,
NCR: 4 cities and 1 municipality.

Random selection was done to determine which among the barangays will be included in the sampling population. The sample size for each barangay included in the sample was 100. Two stools were collected from each of these 100 persons in the sample. The choice of households to be sampled was done by systematic sampling. In Maguindanao, the five barangays in five municipalities were surveyed as recommended by the regional secretary of the Department of Health based on accessibility and peace and order situation of the place.

As in all the phases of the survey, the households were selected in a systematic manner. A masterlist of all the households in the barangay was used as sampling frame in the selection of the households. A total of 274 subjects were required for each barangay included in the sample. Two stool samples were required from each eligible member (referring to those whose age is two years and above) in the selected households.

Sample size was computed based on the prevalence rates from the 1994 World Bank-assisted Philippine Health Development Program. A 50% adjustment added to the original computation for sample size was made for the cluster sampling design and another 10% for nonresponse making the Luzon survey sample size (Phase 3) 4000 and the Maguindanao survey (Phase 4) 1370. A total of 274 individuals were required for each barangay in Maguindanao and 100 individuals per barangay in the Luzon survey.

The study was carried out under the close supervision of the Department of Health officials and personnel assigned in the selected areas. Informed consent of participants was obtained before they submitted stool specimens for Kato-Katz. Survey results were turned over to the municipal health officers of concerned municipalities for management of those found positive for any helminthic infection.

### 2.2. Parasitological Survey and Statistical Analysis

Kato-Katz thick smear examination was used in diagnosing *Schistosoma japonicum *and detecting the presence of eggs of other parasites [7]. Two stool samples collected on two separate days were processed from each individual included in the sample. Aside from schistosome eggs, soil-transmitted helminthes (i.e., *Ascaris lumbricoides*, hookworm, and *Trichuris trichiura*) and food-borne trematodes (e.g., heterophyids, *Taenia* spp., *Paragonimus westermani*, and echinostomes) were recorded in the survey. Both soil-transmitted helminthes and food-borne trematode infections are widespread in Southeast Asia [8, 9].

Prevalence for each province was computed by dividing the number of positives by the total number of individuals whose stools were examined from the five barangays per province.

## 3. Results

Eight provinces were covered in the survey including the National Capital Region. The average response rate at 73.02% was the highest among the four phases with the Mindanao survey second at 70.9% and the Visayas survey last at 32.2%. Maguindanao registered a 45.2% response rate in spite of the peace and order problem in the province.

Seven helminthic infections were reported in Luzon and Maguindanao, namely, schistosomiasis, ascariasis, trichuriasis, hookworm infection, heterophyidiasis, taeniasis, and paragonimiasis.

**Figure 1:**
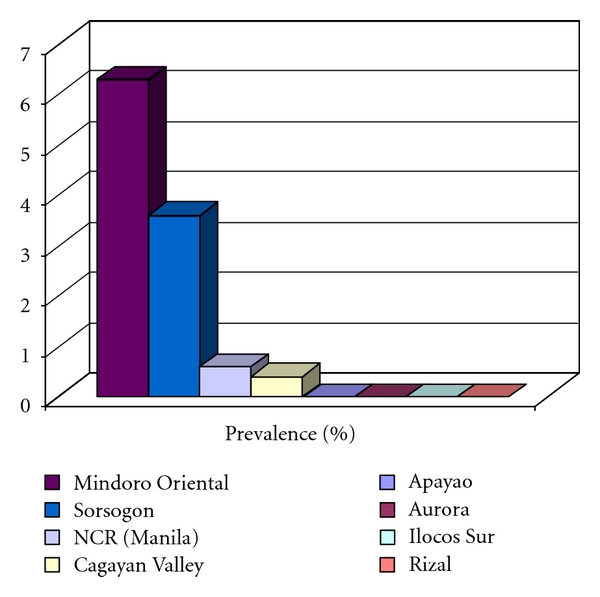
Prevalence of schistosomiasis in Luzon, 2007.

Figure 1 shows the prevalence of schistosomiasis in Luzon with Mindoro Oriental ranking first at 6.3% followed by Sorsogon at 3.6%. Cases were also reported from NCR. These turned out to be migrants from Cagayan Valley.

**Figure 2:**
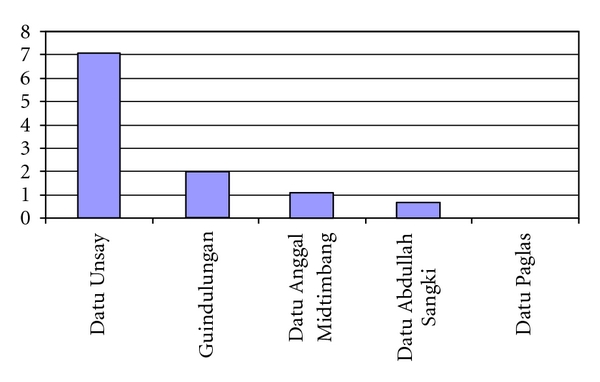
Prevalence of schistosomiasis in Maguindanao by municipalities, 2008.

Figure 2 shows that four out of five municipalities surveyed in Maguindanao were positive for schistosomiasis with prevalence ranging from 1.1% to 7.1% with an average of 1.8%.

**Figure 3:**
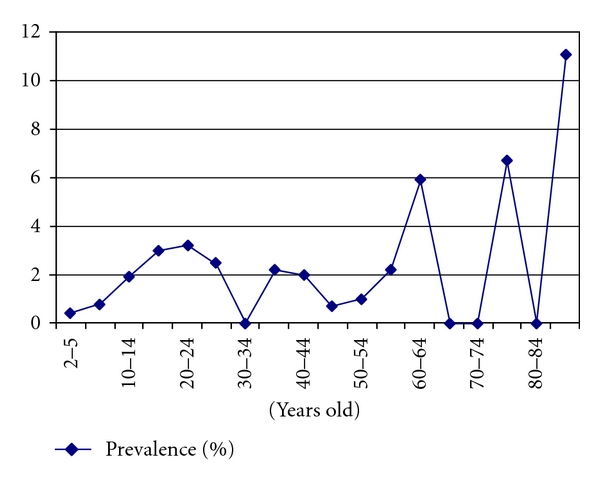
Age-specific prevalence of schistosomiasis in Luzon, 2007.

The age distribution curve in Luzon found in Figure 3 shows older age groups with higher prevalence compared to the younger age groups. Peaks of prevalence are noted among the 20–24, 50–59, and 75–79 age groups. In Maguindanao, highest prevalence is noted among the 50–54 age group. Sex-specific prevalence is almost four times higher in males compared to females in Luzon, whereas in Maguindanao the difference is not that high with males at 2.4% and females at 1.6%.

Aside from schistosomiasis, STH and food-borne helminthes were recorded in Luzon and Maguindanao.  Table 1 shows the prevalence of these other helminthes.

Age distribution curves for STH in Luzon indicate peaks not only for the younger age groups but older age groups as well. Prevalences are similar for both sexes.

## 4. Discussion

Maguindanao and Marawi City are the two places in the Autonomous Region of Muslim Mindanao (ARMM) that were included in the Mindanao survey or Phase 1 of the National Baseline Prevalence Survey of Schistosomiasis Project. Maguindanao was chosen since it has consistently been considered as a highly endemic province for schistosomiasis. In 1997, it was ranked first in the list of schistosomiasis endemic provinces in the Philippines with a prevalence rate of 18.9%. It was grouped with Agusan del Sur and Lanao del Norte, both provinces from Mindanao as the high prevalence provinces. In 2005, it continued to maintain its status as a high prevalence province with a prevalence rate of >10% together with Agusan del Sur, Butuan City, and the new endemic province of Negros Occidental.

In the present survey, the prevalence rate recorded for Maguindanao after averaging the prevalence rates from the 5 municipalities surveyed was 1.8%. Although it took three years to finally complete the survey in Mindanao with the addition of the Maguindanao data, it would be interesting to see how the trend changes for the regions in Mindanao with the inclusion of the Maguindanao results. For example, in the 2005 survey, Region 13 or Caraga ranked first in prevalence of schistosomiasis followed closely by Region 11 and Region 10, but with the inclusion of the Maguindanao results ARMM moved to second place with a large edge over Regions 11 and 10 (Table 2).

In terms of ranking by province, Maguindanao ranks second after Agusan del Sur bumping off Bukidnon from second place. Table 2 shows the ranking of the other endemic provinces of Mindanao. The Maguindanao results did not change the sex distribution and the age distribution of the disease.

With the inclusion of the results from Luzon (Phase 3) and Maguindanao (Phase 4), the ranking of the provinces based on prevalence of schistosomiasis changed and the overall distribution of the disease became clearer [5]. Mindoro Oriental from Luzon showed the highest prevalence followed by Agusan del Sur from Mindanao and then Sorsogon, another province from Luzon, coming in third. While the outcome of the study confirmed previous findings about the situation of schistosomiasis in the Philippines, new information have been generated which are of great import in the updating of the schistosomiasis control program. The final ranking of the provinces based on the prevalence of schistosomiasis is shown in Table 3. The Philippine Department of Health in 2007 listed 28 schistosomiasis endemic provinces most of which are found in Mindanao [2]. The same distribution of the disease is maintained in this study with 60.8% of the 23 endemic provinces in Mindanao, 21.7% in the Visayas, and 17.4% in Luzon. 

Table 4 lists the other provinces surveyed which did not yield cases of schistosomiasis during the period of survey. Response rate in this survey was 45.2% which is relatively low. The barangays surveyed were pre-selected because they had lesser problems in peace and order. The prevalence of schistosomiasis could be higher in the excluded barangays because the peace and order problems could have disrupted or even altogether precluded the delivery of health care in these communities.

**Figure 4:**
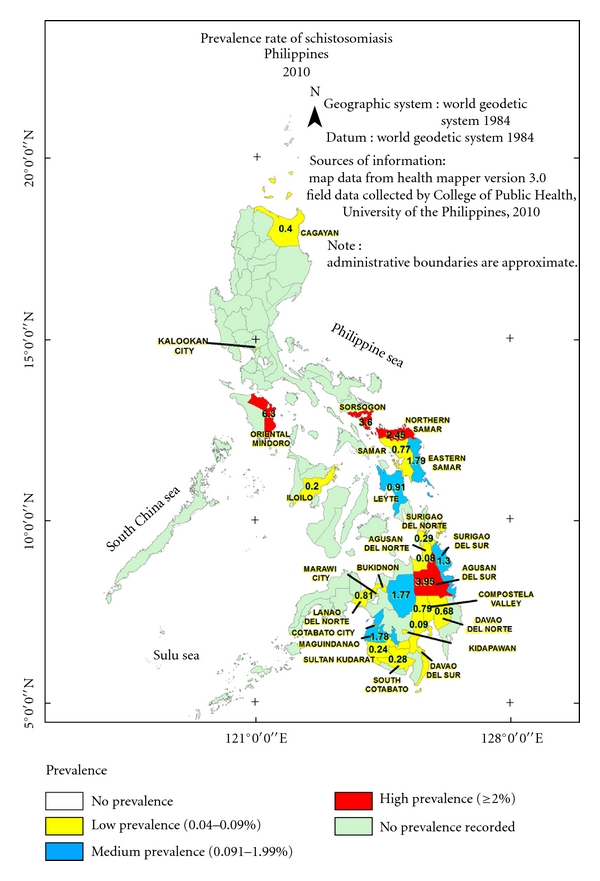
Field data collected by the schistosomiasis research team, College of Public Health, University of the Philippines Manila 2005–2008.

The map in Figure 4 shows that high prevalence areas are concentrated more in Luzon and the Visayas compared with Mindanao. The high prevalence noted in Mindoro Oriental can be attributed to the high transmission rate brought about by the continuous rainfall and the subsequent floods that facilitate human-parasite contact. Heavy rains frequently cause the Naujan Lake in Mindoro Oriental to overflow exposing people to contaminated waters. The ease with which people move between Sorsogon and Northern Samar could facilitate the exchange and buildup of cases in these two adjacent provinces. It should be noted that the Maguindanao survey was conducted three years after that of Mindanao. During this interval, several rounds of mass treatment have already been conducted to bring down the prevalence of schistosomiasis to its level of 1.8% in 2008. Comparison of the prevalence of the disease in Maguindanao with that of the other provinces in Mindanao should be interpreted with caution. 

Agusan del Sur used to rank first in prevalence of schistosomiasis, and flooding caused by frequent overflowing of the Agusan Marsh has been blamed for this. Overall however, the situation in Mindanao has improved with many of the endemic areas enjoying significant reduction in disease prevalence. Mindanao has been the beneficiary of numerous local and international projects aimed at improving the health and economic situation in the island. Most of the efforts to address schistosomiasis used mass chemotherapy which successfully brought the prevalence down but is not expected to eliminate the disease. There is therefore a need to augment mass chemotherapy with other measures such as snail control, environmental sanitation, and health education that could bring more enduring outcome. 

In the Visayas, Region 8 continues to be highest in prevalence with the endemic provinces exceeding the prevalence of those in Mindanao. Visayas also surpasses Mindanao and Luzon with respect to prevalence of ascariasis and trichuriasis. These are indicators of the possible chronic inattention that the region has suffered for a long time. With most of the external and internal assistance being funneled into Mindanao, the region has remained in a dire economic situation. Schistosomiasis, ascariasis, and trichuriasis are considered diseases of poverty spawned by inadequate environmental sanitation, bad personal hygiene, and widespread illiteracy. 

In Luzon, the disease remains in the old endemic provinces like Mindoro Oriental in Region 4B and Sorsogon in Region 5. The cases that were found in Caloocan City, which is located in the National Capital Region, were found to have previously worked as farmers in Cagayan Valley where they could have contracted the disease. The survey also noted cases in the newly discovered endemic province, Cagayan Valley. This new focus is found in the northern tip of Luzon and is considered as one of the hottest areas in the country. In another study led by the senior author, the endemicity of the disease in this new focus was confirmed based on the presence of indigenous cases, infected *Oncomelania quadrasi* snails, and infected animals. The discovery of the disease in Cagayan Valley has raised concerns about the possible spread of the disease from this new site to neighboring provinces. This apprehension seems reasonable given that the snail intermediate hosts can be easily transported to other places during heavy rains and flooding. 

The extensive distribution of schistosomiasis in Mindanao compared to the Visayas and Luzon may be attributed to high degree of connectivity in this large island. Hydrological connectivity through irrigation and river networks coursing through various municipalities and provinces in Mindanao can most likely transport snails into previously nonendemic areas. While this flow is unidirectional in nature, socioeconomic connectivity is more diffusive such as movement of human or animal labor or trading of fertilizer from adjoining villages [10]. Employment opportunities from huge developmental projects attract laborers from both endemic and nonendemic villages transporting the infection to the host village or bringing home the infection to their village of origin. Even small work opportunities move people from one area to another. Exchange of animal labor, for example, carabaos in farming, can produce the same effect not only in transporting the disease but also the snail intermediate hosts that could possibly adhere to the skin and feet of the animals when they wallow in the mud or in the water. Movement of people has been blamed largely for the transport of schistosomiasis in new endemic sites [11]. 

The limited distribution of schistosomiasis in the eastern part of Luzon, Visayas, and Mindanao confirms the demand of the parasite and its snail intermediate host for specific environmental parameters, particularly rainfall [12]. In the Philippines, these provinces have continuous rainfall all throughout the year. The discovery of the disease however in Cagayan Valley and Negros Occidental, which are both known to have pronounced wet and dry seasons, has put the traditional profile of a schistosomiasis endemic province (e.g., Type IV climate with rainfall more or less evenly distributed throughout the year) in question. Schistosomiasis could remain where it is right now. But given the high mobility of the population not to mention hydrological connectivity, it could just be a matter of time before it is discovered in other unexpected provinces and this continues to worry authorities [11]. 

The current cross-sectional survey confirmed the schistosome-endemic provinces in Luzon, the Visayas and Mindanao, although the rates are much lower for reasons which have been explained previously [5]. That schistosomiasis is a highly focal disease unlike other fecal borne-diseases such as ascariasis and trichuriasis that affect the general population has been demonstrated in this study [13, 14]. The lower prevalence rates generated from this study compared with previous surveys should not lower the guard of control authorities but instead prompt them to dig deeper to unveil the more detailed situation of the endemic areas through more intensive local surveys to identify “hot spots” of the disease [15]. 

As expected, the survey showed other fecal-borne helminthiases that affect people in endemic areas aside from schistosomiasis. Visayas recorded the highest prevalence in soil-transmitted helminthiases such as ascariasis and trichuriasis, considered as concrete proof of widespread neglect of environmental sanitation, poor personal hygiene and illiteracy. In Luzon, the regular pattern of higher prevalence rates among the younger age groups was not easily seen compared with the same pattern in the Visayas and Mindanao. Age distribution curves from the different provinces surveyed in Luzon showed higher peaks in older age groups compared with the younger age groups. That ascariasis and trichuriasis are highly prevalent not only among the younger age groups but in older people as well may be a real cause for concern. This could mean a breakdown in environmental sanitation such as lack of toilets and lack of water supply that could affect both young and old and prevent the old from practicing whatever personal hygiene they may be aware of. 

While these two helminthic diseases affect children mostly, hookworm infection registered a higher prevalence among the working age groups. It is possible that farmers and fishermen are faced with the double morbidity of schistosomiasis and hookworm infection both of which have the same mode of infection, that is, percutaneous infection that could further aggravate their health situation [16, 17]. The prevalence of hookworm infection recorded in this survey may not show the real picture of the disease in the provinces surveyed since there was no rapid examination protocol used to detect hookworm per se. Admittedly, the present method used in the survey could have resulted in major underestimates of hookworm prevalence. The hookworm data are nevertheless too valuable to dispense with since they offer important basis for intervention programs like age and sex distribution and cooccurrence with schistosomiasis. 

That Mindanao presented the highest prevalence of heterophyidiasis confirms suggestion that this parasitic infection is an emerging problem in the island. The proliferation of backyard aquaculture using night soil as cheap fertilizer compounded by the locals' fondness for broiled fish ensure the perpetuation of the parasite's life cycle. Cases of heterophyidiasis were also found in two provinces in Luzon and five provinces in the Visayas. In Luzon, taeniasis was found to be more widespread affecting five provinces with the highest prevalence recorded in Apayao. It was also noted in the Visayas with an average prevalence of 0.5% and affecting more men than women. The increasing fondness for partially roasted goat's meat exposes the men folk to the disease. Sporadic cases of paragonimiasis and echinostomiasis were also noted in the survey. 

Results of the study ascertain the wisdom of an integrated helminth control that would include mass chemotherapy, environmental sanitation and health education and promotion that would change people's behavior and lessen their exposure to these parasitic infections [6, 17, 18]. 

This national baseline survey for schistosomiasis, which has for the first time utilized a well-defined sampling design to accommodate the focal and nonrandom distribution of schistosomiasis, updated the magnitude and distribution of not only schistosomiasis but also other fecal-borne helminthic infections. This information can be used as basis for reallocation of resources depending on the disease situation where the Visayas could hopefully be assigned a bigger chunk of the pie. It is also hoped that in the future, prevalence surveys that will be done to monitor or evaluate success of intervention programs would employ the same sampling design, for example, stratified two-step systematic cluster sampling design for reasons explained in the paper. 

## Figures and Tables

**Table 1:** Prevalence of other helminthic infections in Luzon and Maguindanao, 2007 and 2008.

Other helminthic infections	Luzon	Maguindanao
Ascariasis	18.4	15.3
Trichuriasis	20.8	18.1
Hookworm infection	4.6	0.3
Heterophyidiasis	0.1	0.8
Taeniasis	0.7	0
Paragonimiasis	0	0.1

**Table 2:** Ranking of provinces in Mindanao according to prevalence of schistosomiasis, 2005 and 2008.

Rank	Province	Prevalence	Region
1	Agusan del Sur	4.0	Caraga
2	Maguindanao	1.8	ARMM
3	Bukidnon	1.8	10
4	Surigao del Sur	1.3	Caraga
5	Lanao del Norte	0.8	10
6	Davao del Norte	0.8	11
7	Compostela Valley	0.7	11
8	Cotabato + Kidapawan	0.5	12
9	Marawi City	0.4	ARMM
10	Surigao del Norte	0.3	Caraga
11	South Cotabato	0.3	12
12	Sultan Kudarat	0.2	12
13	Davao del Sur + Digos	0.1	11
14	Agusan del Norte	0.1	Caraga

**Table 3:** Prevalence of schistosomiasis in endemic provinces 2005–2008.

Rank	Name of province	Prevalence (%)	95% CI	Region
1	Mindoro Oriental	6.3	4.40–8.80	4B
2	Agusan del Sur	3.9	3.10–5.00	Caraga
3	Sorsogon	3.6	2.20–5.70	5
4	Northern Samar	2.4	1.50–3.70	8
5	Eastern Samar	1.8	1.00–2.90	8
6	Maguindanao	1.8	0.90–3.20	ARMM
7	Bukidnon	1.8	1.00–2.60	10
8	Surigao del Sur	1.3	0.70–2.20	Caraga
9	Leyte	0.9	0.40–1.70	8
10	Lanao del Norte	0.8	0.40–1.50	10
11	Davao del Norte	0.8	0.30–1.50	11
12	Western Samar	0.8	0.10–2.76	8
13	Compostela Valley	0.7	0.30–1.50	11
14	Caloocan	0.6	0.00–3.20	NCR
15	Cotabato + Kidapawan	0.5	0.20–1.10	12
16	Marawi City	0.4	0.10–1.50	ARMM
17	Cagayan Valley	0.4	0.10–1.60	2
18	Surigao del Norte	0.3	0.10–0.90	Caraga
19	South Cotabato	0.3	0.00–1.00	12
20	Sultan Kudarat	0.2	0.00–0.90	12
21	Iloilo	0.2	0.00–0.70	6
22	Davao del Sur + Digos	0.1	0.00–0.50	11
23	Agusan del Norte	0.08	0.00–0.40	Caraga

**Table 4:** List of other provinces and cities surveyed in 2005–2008.

Name of province	Region
Zamboanga Sibugay	9
Zamboanga City	9
Camiguin	10
Iligan City	10
Misamis Occidental	10
Misamis Oriental	10
General Santos City	12
Sarangani	12
Capiz	6
Negros Occidental	7
Bohol	7
Negros Oriental	6
Apayao	Cordillera administrative region
Ilocos Sur	1
Aurora	3
Rizal	4-A
Manila	National capital region
Quezon City	National capital region
Pateros	National capital region
Malabon	National capital region
